# Intestinal host–microbe interactions fuel pulmonary inflammation in cigarette smoke exposed mice

**DOI:** 10.1080/19490976.2025.2519699

**Published:** 2025-06-29

**Authors:** Sune K. Yang-Jensen, Nora Näegele, Si Brask Sonne, Louis Koeninger, Marie Pineault, Félix Tremblay, Nanna Ny Kristensen, Lene Bay, Sophie Aubin, Mathieu C. Morissette, Benjamin A. H. Jensen

**Affiliations:** a Department of Biomedical Sciences, Faculty of Health and Medical Sciences, University of Copenhagen, Copenhagen, Denmark; b Department of Human Health, Novonesis A/S, Lyngby, Denmark; c Department of Medicine, Quebec Heart and Lung Institute, Université Laval, Quebec, Canada; d Faculty of Medicine, Université Laval, Quebec City, Canada; e Costerton Biofilm Center, Faculty of Health and Medical Sciences, University of Copenhagen, Copenhagen, Denmark

**Keywords:** Microbiota, gut, lung, gut–lung axis, cigarette smoke, mucus, COPD, bacterial encroachment

## Abstract

The gut microbiota has been implicated in numerous aspects of host health and immune regulation. Specifically, recent studies have linked gut microbes to the pathogenesis of chronic obstructive pulmonary disease (COPD), primarily induced by excessive cigarette smoke, although the underlying mechanisms remain elusive. Here, we investigated the role of gastrointestinal (GI) host–microbe interactions on pulmonary health. Using two distinct means of modulating GI host–microbe relations, we dissected how gut microbes fuel pulmonary inflammation in mouse models of cigarette smoke (CS)-induced lung disease. We found that CS caused profound changes to the colonic mucosa, with reduced mucus and increased bacterial encroachment. Modulating host–microbe interactions using antibiotics and recombinant human β-defensin 2 restricted colonic bacterial encroachment, limiting interactions between host and microbe. These strategies resulted in substantial ~50% decrease in pulmonary neutrophil infiltration following both acute and chronic exposure to CS. The reported findings provide additional evidence of a gut–lung axis, offering novel insight into the role of the gut microbiota in pulmonary immune activation, which could represent a novel avenue for future therapeutic strategies.

## GRAPHICAL ABSTRACT

**Figure:**
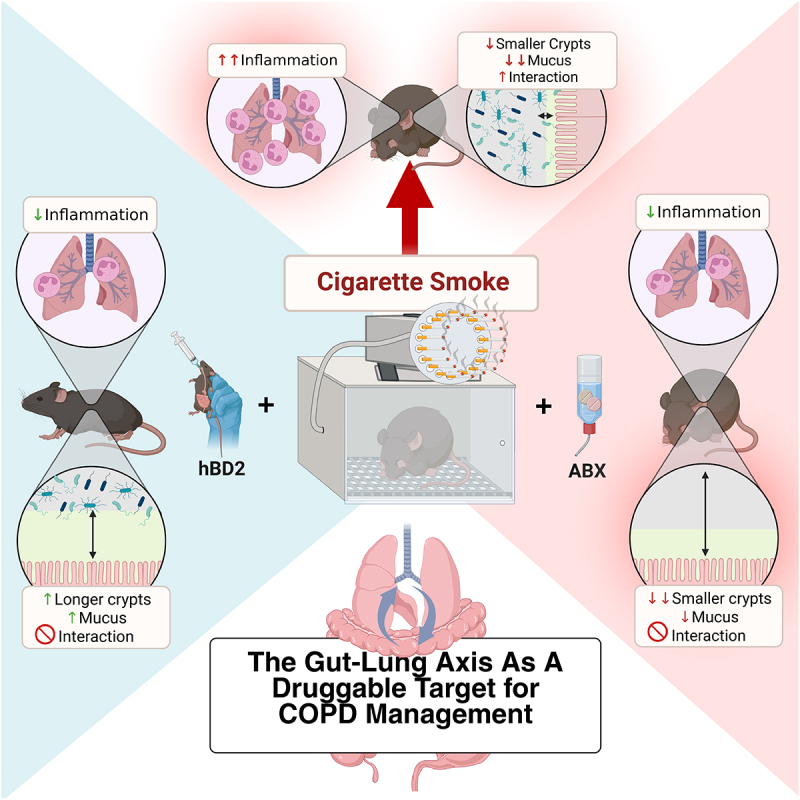

## Introduction

Chronic obstructive pulmonary disease (COPD) is the third leading cause of mortality worldwide, responsible for more than 3.2 million deaths in 2019^1^, posing an immense burden on global health-care systems. COPD is a multifactorial lung disease characterized by pathological alterations of large and small airways as well as lung parenchyma with cigarette smoke (CS) as the primary etiological factor. Patients with COPD remain prone to lung infections and sustained airway inflammation even after smoking cessation^2^. Current therapies focusing on bronchodilation and immunosuppression with corticosteroids provide symptomatic relief but fail to address underlying disease mechanisms, while paradoxically increasing infection risks^3^. This therapeutic impasse has spurred interest in alternative pathways linking mucosal immunity and environmental exposures to COPD progression.

To this end, emerging evidence implicates a dysregulated gut–lung axis in chronic respiratory diseases^4,5^, with particular relevance for COPD, where the gut microbiota has been proposed to causally influence disease development and progression^6–10^. This influence is mediated through several interconnected mechanisms: the production of microbial metabolites such as short-chain fatty acids (SCFAs), the trafficking of immune cells between mucosal sites, and, in cases of barrier dysfunction, the systemic spread of microbial components^11–16^. Crucially, swallowing of pulmonary secretions, debris, microbes, and CS extracts^17,18^ may induce gut barrier impairment and microbial dysbiosis in both patients and animal models^6–9,17^, creating a potential feedback loop where intestinal inflammation exacerbates pulmonary pathology.

Considering the involvement of the gut–lung axis in COPD progression, there is increasing interest in therapeutic strategies that target the gut–lung axis to modulate disease outcomes. Supporting this notion, recent interventions targeting microbial communities, such as antibiotic regimens and host defense peptides (HDPs), have shown promise in alleviating multiple pulmonary disorders^19–22^. However, the specific mechanisms by which gut microbes influence COPD trajectory continue to elude researchers, particularly regarding the spatial dynamics of host–microbe interactions at mucosal interfaces. While antibiotics reduce pulmonary inflammation in COPD models, their long-term use raises concerns about microbial resistance and disruption of gut barrier homeostasis. In contrast, HDPs offer a targeted approach to strengthen mucosal barriers while preserving microbial diversity. These contrasting strategies – microbial eradication versus modulation of host–microbe interactions – provide complementary tools to dissect gut–lung communication mechanisms.

One well-characterized HDP is human β-defensin 2 (hBD2). hBD2 originates from epithelial cells, including enterocytes, following stimulation by pro-inflammatory molecules such as certain cytokines or microbial components. Importantly, *in vitro* studies have shown direct interactions between hBD2 and immune cells, affecting their chemotaxis and maturation^23–25^. Moreover, multiple animal studies corroborate that oral administration of recombinant hBD2 can improve outcomes in various inflammatory conditions of intestinal^26,27^ and extraintestinal^19–21,28^ origin. Given its dual role in protecting against microbial invaders and modulating host immune responses, hBD2 represents a unique tool for investigating host–microbe interactions.

Based on previously reported causality between the gut microbiota and COPD progression, we hypothesized that host–microbe interactions may play an important role in aggravating pulmonary inflammation during CS exposure. To test this hypothesis, we employed orthogonal interventions in either acutely (4 days) or prolonged (5 days/week for 7 weeks) CS-exposed mice. First, we used an orally delivered broad-spectrum antibiotics cocktail to reduce microbial load, thereby reducing the interactions between the host and its microbial inhabitants. Second, we used orally delivered hBD2 to specifically target host–microbe interactions at the intestinal barrier. By comparing the effects of these strategies on gut barrier integrity, microbial ecology, and pulmonary inflammation, we identify host–microbe interaction dynamics as a critical regulator of CS-induced lung pathology.

## Results

### Antibiotics and oral hBD2 administration non-additively ameliorate lung inflammation after cigarette smoke exposure

**Figure 1.**
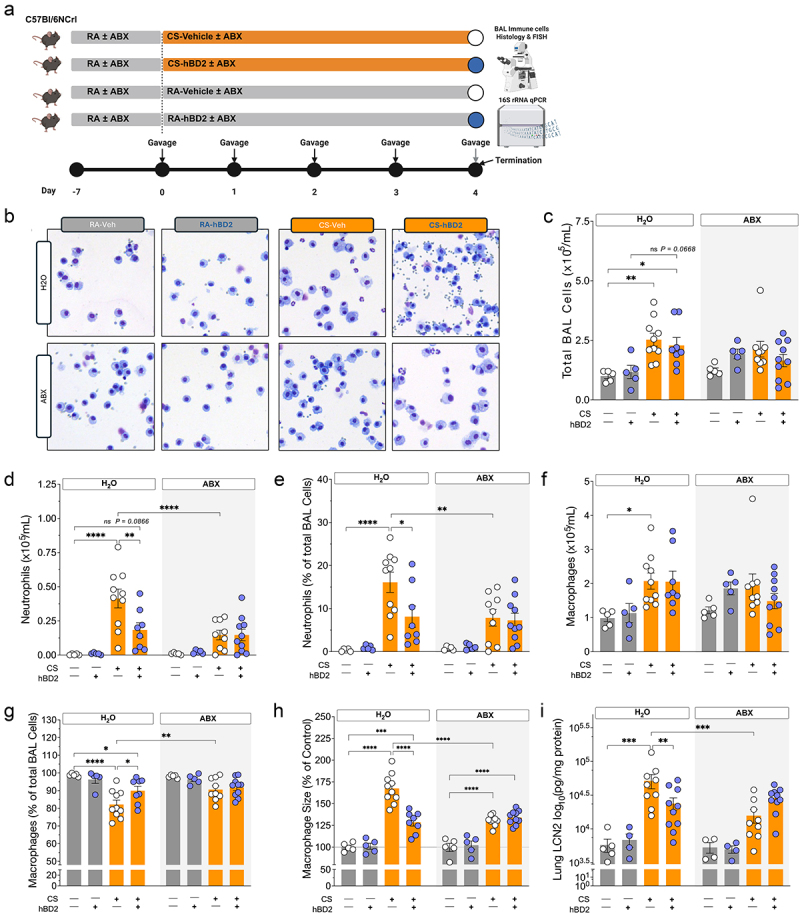
Oral hBD2 and oral antibiotics reduce pulmonary inflammation following acute cigarette smoke exposure. (a) study design depicting the timeline of acute cigarette smoke exposure, oral hBD2, and antibiotic treatments. (b) Representative images of pulmonary immune cells collected by bronchoalveolar lavage (BAL). Images represent the median total BAL cell count per group. (c – h) quantitative analyses of immune populations in BAL samples. (c) total BAL immune cell count. (d) absolute neutrophil count. (e) relative neutrophil count. (f) absolute macrophage count. (g) relative macrophage count. (h) relative macrophage size. (i) pulmonary levels of the neutrophil marker LCN2. Data are presented as mean ± SEM (n = 5 mice per group for room air exposed mice and n = 10 mice per group for cigarette exposed mice. 2 mice in the CS-hBD2-water group had their lungs punctured during dissection, thus no BAL was collected). Select comparisons are shown between groups of interest, all comparisons can be found in the online supplementary material. *p* values were calculated using two-way ANOVA, Tukey’s post hoc test adjusting for multiple comparisons, with a significance threshold of p < 0.05.

**Figure 2.**
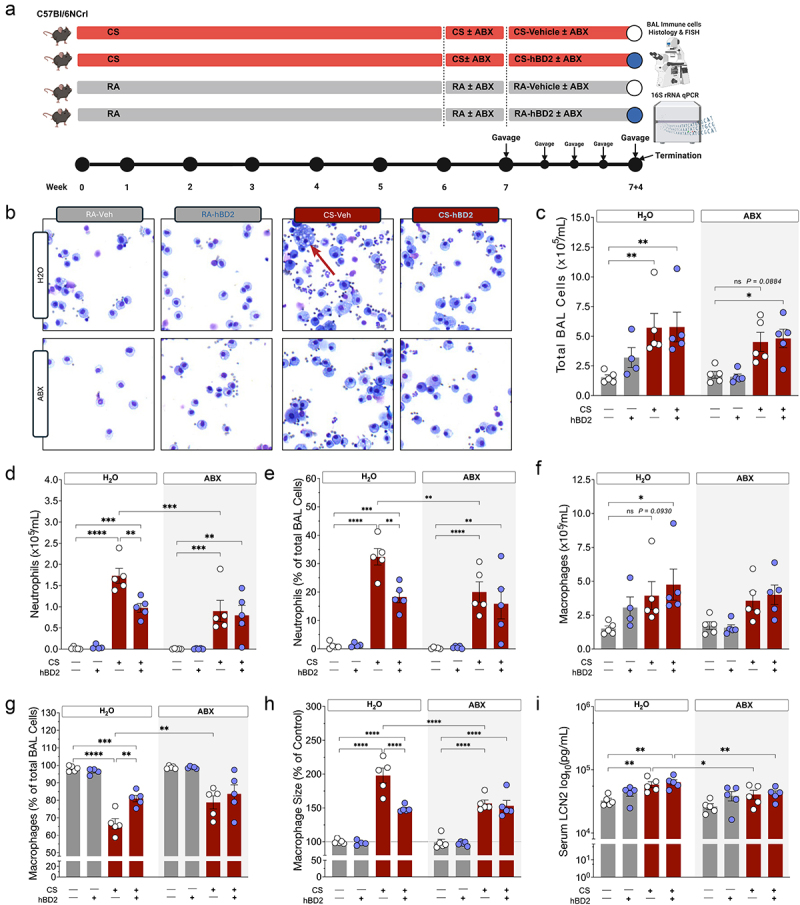
Oral hBD2 and oral antibiotics reduce pulmonary inflammation following chronic cigarette smoke exposure. (a) study design depicting the timeline of chronic cigarette smoke exposure, oral hBD2, and antibiotic treatments. (b) Representative images of pulmonary immune cells collected by bronchoalveolar lavage (BAL). Images represent the median total BAL cell count per group. Red arrow: macrophage foam cell. (c – h) quantitative analyses of immune populations in BAL samples. (c) total BAL immune cell count. (d) absolute neutrophil count. (e) relative neutrophil count. (f) absolute macrophage count. (g) relative macrophage count. (h) relative macrophage size. (i) serum levels of the neutrophil marker LCN2. Data are presented as mean ± SEM (n = 5 mice per group. One mouse in the RA-hBD2-water group had its lungs punctured during dissection, thus no BAL was collected). Select comparisons are shown between groups of interest, all comparisons can be found in Supplemental_Material_1. *p* values were calculated using two-way ANOVA, Tukey’s post hoc test adjusting for multiple comparisons, with a significance threshold of p < 0.05.

It has previously been reported that certain antibiotics and hBD2 can limit neutrophil infiltration after CS exposure in mice^6,21^. However, the combination of these treatments has not been investigated nor has the underlying mechanisms. To elucidate this, we exposed mice to acute or prolonged CS (Figures 1(a) & 2(a)) in addition to two interventions targeting host–microbe interactions. In the acute protocol, mice were exposed to RA or CS for four consecutive days. During this period, hBD2 was delivered via oral gavage 2 hours after each exposure. To diminish gut microbial load, a subset of mice was pre-treated with antibiotics from 7 days before hBD2 administration and CS exposure and maintained throughout the study. In the prolonged protocol, mice were exposed to CS or RA for 7 weeks with or without hBD2 administration the final 4 days, representing an extremely short-term intervention. Mirroring the acute experiment, a subset of mice was pre-treated with antibiotics from 7 days before administration until conclusion of the protocol.

Acute (Figure 1(b,c)) and prolonged (Figure 2(b-c)) CS exposure increased the total number of immune cells present in the bronchoalveolar lavage (BAL) independently of antibiotics or hBD2 administration. CS exposure caused pronounced pulmonary neutrophilia after just 4 days of acute exposure, with ~16% of all BAL cells being neutrophils (<1% in RA exposed mice) (Figure 1(d,e)) – this was starkly increased to ~33% after prolonged CS exposure (Figure 2(d,e)). hBD2 administration significantly reduced neutrophil infiltration by ~50% in acutely exposed mice (Figure 1(d,e)). Despite being a very short-term intervention, hBD2 delivery produced a highly similar ~46% decrease in neutrophil infiltration following prolonged CS exposure (Figure 2(d,e)). Surprisingly, antibiotic treatment in both protocols exerted neutrophilia-lowering effects akin to hBD2 administration – however, the combined intervention strategy did not yield additive protective effects after neither acute nor prolonged CS exposure (Figure 1(d,e) & 2-DE, respectively). hBD2 administration and antibiotic treatment had little effect on total BAL macrophage numbers independently of CS duration (Figures 1(f) & 2(f)). We observed a relative decrease in the BAL macrophage population after acute exposure (Figure 1G) which was more pronounced after prolonged CS exposure (Figure 2(g)), likely as a function of the stark neutrophil infiltration. Thus, the neutrophilia lowering effects of hBD2 and antibiotics partially restored the balance between alveolar macrophages and neutrophils after both acute (Figure 1(g)) and prolonged (Figure 2(g)) exposure to CS. With the resolution offered by the current method of quantification no changes were observed in other immune compartments after acute or prolonged CS exposure (Suppl. Fig. S1A-B).

Pulmonary macrophage size is typically increased in smokers and rodent models of cigarette smoking and associated with reduced macrophage phagocytic ability^29^. CS significantly increased the size of alveolar macrophages in the BAL (Figures 1(h) and 2(h)) with macrophages resembling foam cells after prolonged CS exposure (Figure 2(b), red arrow). Importantly, hBD2 administration and antibiotics effectively rescued this trajectory in the acute (Figure 1(h)) and prolonged (Figure 2(h)) CS exposure models. While acute CS exposure increased local pulmonary levels of the neutrophil degranulation-marker lipocalin-2 (LCN2) (Figure 1I), the short-term protocol did not increase systemic LCN2 levels (Suppl. Fig. S1C). Interestingly, hBD2 and antibiotic administration substantially reduced pulmonary levels of LCN2 (*p* = 0.0078 and 0.0001, respectively, Figure 1I), completely reflecting the reduction of lung neutrophilia. In contrast to the acute exposure, prolonged CS exposure caused systemic low-grade inflammation (Figure 2(i)); however, this was ameliorated by 11 days antibiotic treatment (Figure 2(i)). Combined, these results indicate a less inflammatory pulmonary environment after both hBD2 and antibiotic administration, regardless of CS exposure duration, and hence inflammatory intensity. These data point toward a rapid and restorative effect of the tested strategies. Surprisingly, combining hBD2 with antibiotics following CS exposure did not additively alleviate pulmonary inflammation.

### Oral hBD2 and antibiotics differentially impact fecal and pulmonary microbiota

As HDPs, including hBD2, were first described as endogenous antibiotics with notable antimicrobial functions, at least *in vitro*^24,30,31^, we next examined if hBD2 and antibiotics had similar eradicative effects on gut and lung microbiota by 16S sequencing.

**Figure 3.**
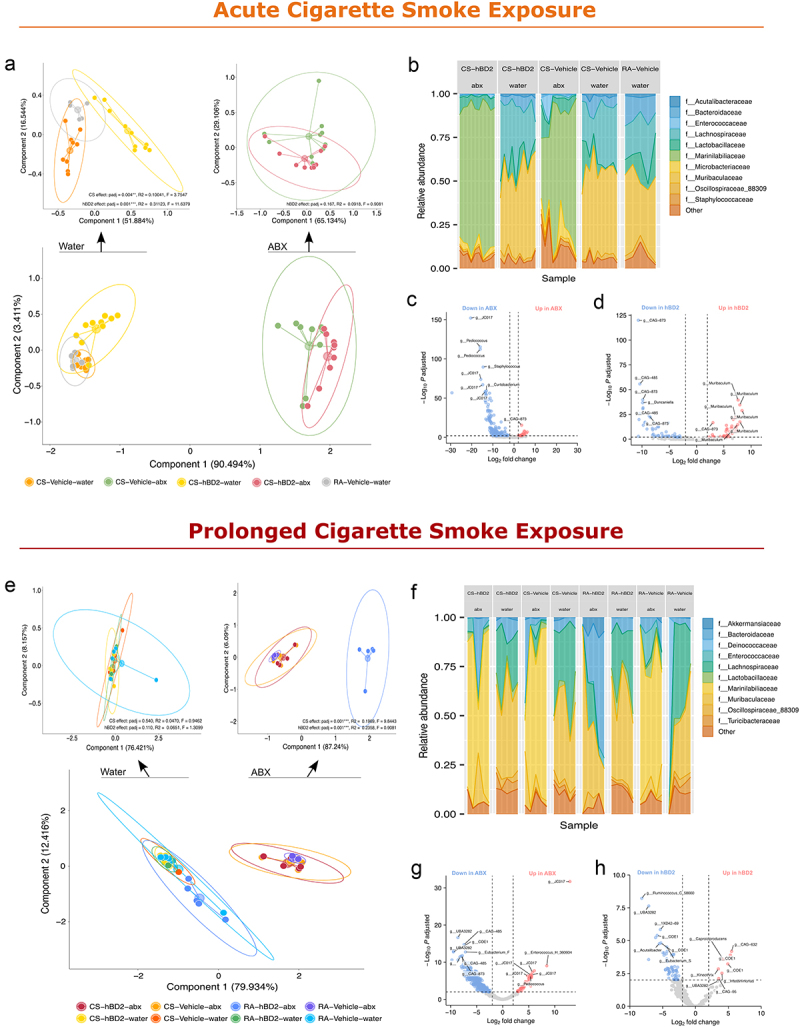
hBD2 and antibiotics distinctly impact the fecal microbiota. (a–d) Fecal microbiota analyses of mice exposed to cigarette smoke (CS) acutely. (a) Beta diversity analysis based on weighted UniFrac distances. Zoom panels show FDR-corrected adjusted p-values, R2, and F statistics from PERMANOVA analyses of water and ABX treated mice. (b) Family-level microbiota composition at the termination day. (c) Differentially abundant taxa associated with antibiotic treatment, shown as a DESeq2 volcano plot, indicating genera primarily found in water and lost upon antibiotic treatment to the left (negative values) and genera primarily found in antibiotic treated mice to the right (positive values). (d) Differentially abundant taxa associated with hBD2 administration, shown as a DESeq2 volcano plot, with genera primarily found in vehicle treated mice to the left (negative values) and genera primarily found in hBD2 administered mice to the right (positive values). (e-h) Fecal microbiota analyses of mice after prolonged cigarette smoke exposure. (e) Beta diversity analysis based on weighted UniFrac distances. Zoom panels show FDR-corrected adjusted p-values, R2, and F statistics from PERMANOVA analyses of water and ABX treated mice. (f) Family-level microbiota composition at the termination day. (g) differentially abundant taxa associated with antibiotic treatment, shown as a DESeq2 volcano plot, indicating genera primarily found in water treated mice and lost upon antibiotic treatment to the left (negative values) and genera primarily found in antibiotic treated mice to the right (positive values). (h) Differentially abundant taxa associated with hBD2 administration, shown as a DESeq2 volcano plot, with genera primarily found in vehicle treated mice to the left (negative values) and genera primarily found in hBD2 administered mice to the right (positive values).

Notably, antibiotics, not hBD2, were the main driver of variation (>90%) in fecal microbiota samples as assessed by weighted UniFrac distances, demonstrating clear segregation between antibiotic and non-antibiotic groups (Figure 3(a,b)) and sharply reduced alpha diversity (Shannon index, Suppl. Fig. S2A), in acute CS-exposed mice. In contrast, hBD2 only induced subtle changes to the microbial community structure (Figure 3(a,b)). However, in water treated mice, FDR-corrected PERMANOVA analysis revealed that both acute CS exposure and hBD2 administration had a significant impact on beta-diversity (Figure 3a, p.adj = 0.004 & p.adj = 0.001, respectively) that was not caused by differences in dispersion. Additionally, antibiotic-induced eradication of the gut microbiota resulted in loss of hBD2-mediated effects on gut microbiota modulation (p.adj = 0.167). We next used DESeq2 adapted for microbiota analysis^32,33^ to determine differential taxa abundance and identified taxa altered by the interventions. Differential abundance analysis confirmed the dissimilar effect of hBD2 and antibiotics on fecal microbiota following acute CS exposure (Figure 3(c,d)), with a profound effect imposed by antibiotics (Figure 3(c)) and comparably less convincing effect of hBD2 administration (Figure 3(d)). Interestingly, while antibiotics had little effect on the pulmonary microbiota after acute CS exposure (Suppl. Fig. S2B-F), we did observe hBD2induced alterations on beta-diversity independently of antibiotics treatment (Suppl. Fig. S2D), suggesting oral hBD2 administration can modulate pulmonary microbes, despite not being detected in neither serum nor lung samples by LC-MS analysis (Suppl. Fig. S3A-B).

Consistent with our findings after acute exposure, 11 days of antibiotic treatment remained the main driver of gut microbial changes in beta-diversity (~80% explained variation; Figure 3(e)), alpha-diversity (Suppl. Fig. S2G), and microbial composition (Figure 3(f)) after prolonged CS exposure. PERMANOVA analysis of the weighted UniFrac distances revealed that, in contrast to a prophylactic acute setting, the short administration period of hBD2 (4 days) after prolonged CS exposure (7 weeks) diminished the impact of the HDP on community beta-diversity (Figure 3(e)). Surprisingly, we observed a statistically significant effect of hBD2 in antibiotic-treated mice (p.adj = 0.001). However, this effect was mainly driven by the RA-hBD2 group receiving antibiotics which, across measures (Figure 3(e,f)), resembled water treated mice. While this may be confounded due to the experimental setup of one cage per group, deeper interrogation of this phenomenon revealed that the mice in question had moderately high bacterial loads despite antibiotic treatment, in contrast to other antibiotic treated mice (Suppl. Fig. S4), substantiated by detectable bacterial signal in our fluorescence *in situ* hybridization (FISH) assay (Figure 5), as detailed below. Despite this finding, the effects of hBD2 on fecal microbiota composition were highly dissimilar to antibiotic-treatment, with different microbes and smaller effects observed following differential abundance analysis (Figure 3(g,h)). When examining the pulmonary microbiota after prolonged CS exposure, we did, akin to the findings from acute protocol, not observe notable effects of antibiotics (Suppl. Fig. S2H-J). Yet, just 4 days of hBD2 administration were sufficient to induce beta-diversity changes independently of antibiotic-treatment, as assessed by PERMANOVA on weighted UniFrac distances (water: p.adj = 0.033, abx: p.adj = 0.015). Still, the detected alterations in the pulmonary microbiota composition by any of the microbiota-targeted interventions were generally less stark compared to those observed in fecal samples (Suppl. Fig. S2K-L).

Combined, these data indicate that the fecal and pulmonary microbiota composition were distinctly modulated by hBD2 and antibiotics, corroborating a dissimilar mode of action of the two interventions. Importantly, while antibiotic treatment ameliorated gut microbial loads with minimal impact on the pulmonary microbiota composition, hBD2 administration modulated both microbial niches and preserved microbial load, despite not being detectable in circulation or in lung tissue. Thus, the mechanism behind the reduction of pulmonary neutrophilia by hBD2 and antibiotics are likely distinct and may be mediated by other factors than local modulation of microbial communities.

### Cigarette smoke alters gut morphology and colonic mucus production

It has previously been reported that CS alters intestinal tight junctions^6,34,35^ and that antibiotics not only deplete the gut microbial communities but also alter intestinal tissue homeostasis^36–38^. Since hBD2 was undetectable, or close to the limit of detection in circulation, lungs, and intestines (Suppl. Fig S3), we speculated that hBD2 and antibiotics may have a local effect within the gut that translates to an altered pulmonary environment.

**Figure 4.**
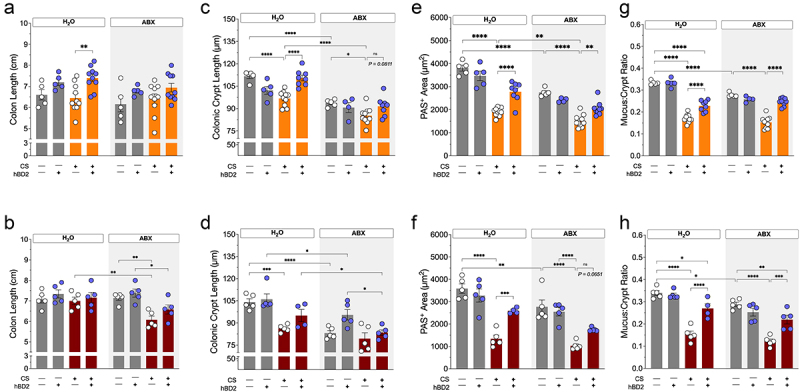
hBD2 counters cigarette smoke- and antibiotics-induced colonic alterations. (a, b) Colon length in acute (a) and prolonged (b) cigarette smoke (CS) protocols. (c, d) Colonic crypt length after acute (c) and prolonged (d) CS exposure. (e, f) Colonic crypt mucus area (PAS^+^ area, μm^2^) shown as the average of 9 full-length crypts per mouse after acute (e) and prolonged (f) CS exposure. (g, h) Colonic PAS^+^ area-to-total crypt area ratio after acute (g) and prolonged (h) CS exposure. Data are presented as mean ± SEM (acute: *n* = 5 mice per group for room air exposed mice and *n* = 8–10 mice per group for cigarette exposed mice. Prolonged: *n* = 4–5 mice per group). Select comparisons are shown between groups of interest, all comparisons can be found in Supplemental_Material_1. Statistical significance was determined by two-way ANOVA, Tukey’s post hoc test adjusting for multiple comparisons, with a significance threshold of *p* < 0.05.

Turning our attention toward the intestines, we observed clear macroscopic effects of antibiotic treatment. Mice receiving antibiotics had enlarged small intestines with pronounced swelling of small intestinal segments and cecum (Suppl. Fig. S5) and diarrhea, independently of CS exposure duration or administration regimen, which was not observed in water treated mice administered with hBD2. This supports the notion of hBD2 and antibiotics having dissimilar modes of action. During acute CS exposure, hBD2 delivery resulted in significantly longer colons of mice maintained on regular drinking water, although less apparent in mice with a blunted gut microbiota following antibiotic treatment (Figure 4(a)). Similar traits were observed in RA-exposed mice. During prolonged CS exposure, the combination of CS and antibiotics had additive effects on colon shortening (Figure 4(b), *p* = 0.0022).

Microscopic examination of the intestines revealed that CS and antibiotics had widespread and often additive effects on gut morphology of the large intestine (Figure 4(c,d)). Acute CS exposure and antibiotics reduced the depth of colonic crypts of the proximal large intestine, with the combination displaying an additive effect (Figure 4(c), *p* < 0.0001). Interestingly, hBD2 administration completely protected against CS-induced colonic crypt shortening, although only in mice not receiving antibiotics. After prolonged CS exposure, crypt shortening became more pronounced and was further aggravated by antibiotics; however, the crypt recovery by short-term hBD2 administration was lost (Figure 4(d)).

Given the apparent impact on the colonic epithelium, we decided to investigate the colonic mucus machinery. However, antibiotics greatly impaired the density of the colonic mucus layer (Suppl. Fig. S6), with swelling of colonic sections reflecting the wide increase in intestinal wet weights we observed in antibiotic treated mice (Suppl. Fig. S5), supporting recent literature on antibiotics-induced mucus defects^39,40^. Therefore, measuring mucus thickness by traditional means proved challenging, prompting us to focus on the crypt-residing mucus-producing goblet cells. Here, it became evident that both CS and antibiotics greatly hampered the production of neutral mucins from goblet cells, with additive effects in both acute and prolonged CS exposed mice (Figure 4(e,h)). Fascinatingly, hBD2 administration prominently rescued the CS-induced reduction of colonic mucus in both conventional and antibiotic treated mice (Figure 4(e,h)), independently of duration of CS exposure, suggesting a rapid protective, restorative effect via direct hBD2–host interactions. Despite being unaffected by hBD2 in an acute CS exposure setting, hBD2 increased expression of *Muc2* in a prolonged exposure setting (Suppl. Fig. S6). This is in accordance with a recent paper, suggesting that hBD2 may improve *Muc2* expression, albeit in diet-induced obese mice^41^.

As colonic crypts were affected by CS and antibiotics, and previous studies have reported an effect of CS on intestinal barrier function targets, including tight-junction proteins (TJPs)^6,34,35^, we analyzed colonic gene expression of key genes involved in barrier integrity. CS altered gene expression of TJPs and host-defense genes in mice exposed to CS both acutely and over prolonged periods (Suppl. Fig. 7), in a fashion hinting toward a counter mechanism against acute CS-induced gut barrier damage that diminishes with prolonged CS. Mirroring the patterns observed from our measurements of pulmonary immune cells (Figure 1), hBD2 and antibiotic administration showed highly similar normalizing effect on TJPs, although only in acutely CS exposed mice. In mice exposed to CS over long time, gene expression of TJPs was reduced, supporting published literature^6,34,35^, yet any effect of hBD2 and antibiotics were lost, indicating CS-induced intestinal changes beyond therapeutic capacities of our interventions.

Taken together, these data highlight the detrimental impact CS and antibiotics have on murine intestines. Both CS and antibiotics alone noticeably perturb gut health, often with additive effects. In contrast, hBD2 administration did not induce any measurable gut perturbations. In fact, hBD2 appears to rescue or prevent some detrimental effects of CS and antibiotics.

### Limiting colonic bacterial encroachment improves pulmonary inflammation

**Figure 5.**
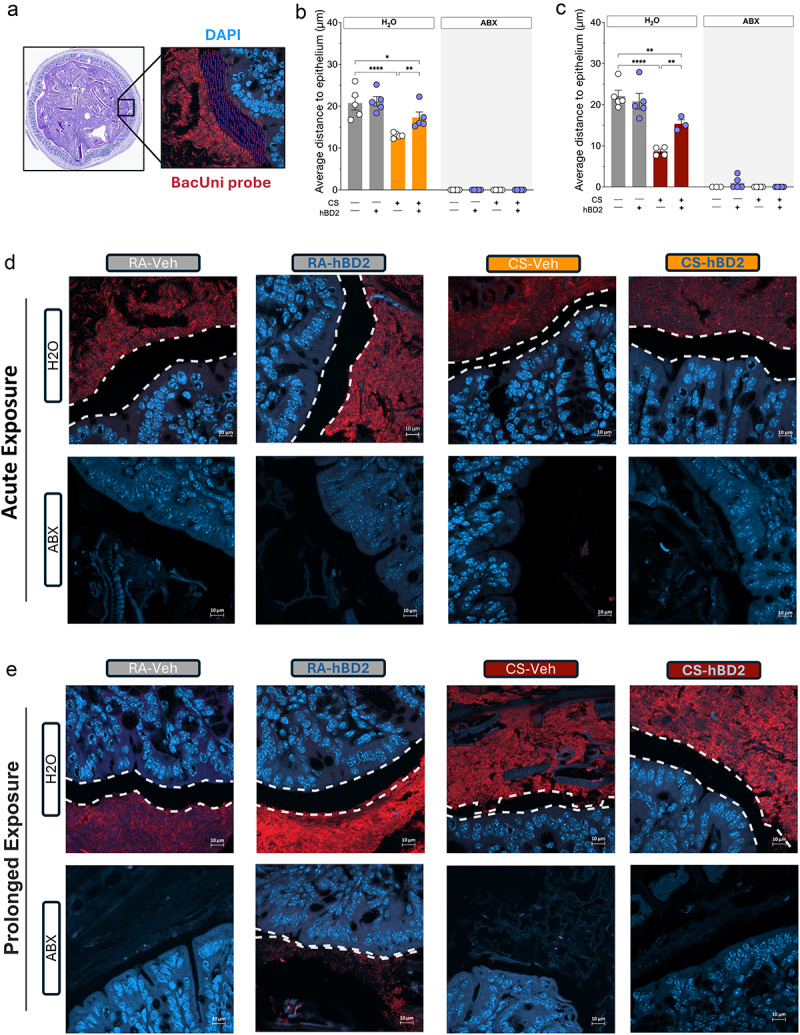
Oral administration of hBD2 increases distance between host and microbe. (a) Schematic outline of PNA-FISH approach, with nuclear DAPI (blue) stain and staining of bacteria with a pan-bacterial BacUni PNA-FISH probe (red). Distance between host and microbes were measured using the automatic measurement function in image-Pro Premier 9.2 giving distances between 100–300 points per area, from three separate areas per section per mouse. (b, c) Average distance between host intestinal epithelium and luminal bacteria in mice exposed to cigarette smoke (CS) acutely (b) and after prolonged exposure (C). (d) PNA-FISH images of distances between host and microbe for acutely CS exposed mice (top) and following prolonged CS exposure (bottom). Images represent group medians. Scalebars, 10 µm. White dotted lines indicate distance between host and microbiota from where measurements were taken. Data are presented as mean ± SEM (*n* = 5 random mice per group per experiment). Select comparisons are shown between groups of interest, all comparisons can be found in Supplemental_Material_1. Statistical significance was determined by two-way ANOVA, Tukey’s post hoc test adjusting for multiple comparisons, with a significance threshold of *p* < 0.05.

Other HDPs have been shown to spatially segregate the microbiota and the intestinal epithelium^42,43^, essentially reducing the interactions between host and microbe. We thus speculated that hBD2 exhibited similarly traits upon CS exposure. To investigate this hypothesis, we analyzed Methacarn-fixated colonic sections stained by a universal bacterial PNA-FISH probe (Figure 5(a)). CS notably decreased the distance of the bacteria to the colonic epithelium by almost 50% after just 4 days of exposure (Figure 5(b-d)). This was recapitulated in mice exposed to CS over prolonged periods of time (Figure 5C+E) in which the distance was >50% decreased. Interestingly, hBD2 administration increased the distance of the microbes to the epithelium considerably, irrespective of CS exposure duration, but only in mice with an already perturbed gut barrier. This was paralleled by improved mucus production, indicating that a healthier mucus layer capable of retaining HDPs^31,44^, may contribute to an increased lysis zone capable of keeping gut microbes at arm’s length and limiting host-microbial interactions. Antibiotic treatment completely ameliorated any bacterial signal, supporting our microbiota analyses (Figure 3, Suppl. Fig. S2, Suppl. Fig. S4), in all but the few previously mentioned mice from the RA-hBD2 group. These same mice had surprisingly high bacterial abundance despite antibiotic treatment (Suppl. Fig. S4), which could explain this phenomenon. Nonetheless, the bacteria in these mice were exceptionally close to the gut epithelium (Figure 5(c,e)), potentially due to the antibiotic-induced disruption of the mucus layer as detailed above. These findings indicate that CS impairs colonic mucus production and allows gut microbes to approach the epithelium, facilitating enhanced host–microbe interactions. Restricting these interactions by enhancing mucus production and thereby increasing the distance between host and microbe or ameliorating the gut microbes all together, as seen by antibiotic treatment, may in return dampen the inflammatory milieu in the airways following CS exposure, irrespective of microbiota modulation.

## Discussion

With this study, we propose that limiting host–microbe interactions at the gut mucosal surface has beneficial effects on pulmonary inflammation in mouse models of acute and prolonged CS exposure. To remarkably similar degree, the two means of modulating host–microbe interactions we explore here, namely oral delivery of the recombinant human HDP, hBD2, and broad-spectrum antibiotics, were capable of alleviating lung inflammation. Although distinct mechanisms are at play, the two strategies ultimately yield similar results – i.e. less directly contact between with the host and its gut microbiota. We show that antibiotic treatment and oral hBD2 reduce pulmonary neutrophil infiltration and alveolar macrophage size following acute and prolonged CS exposure. Yet, these effects are not additive. In support of these two strategies having distinct modes of action, mice administered with hBD2 did not exhibit the same overall phenotypical adverse characteristics as mice ingesting antibiotics (e.g., intestinal and cecal swelling, perturbed colon morphology, and diarrhea). Moreover, hBD2 administration preserved microbial load and modulated both pulmonary and gut microbial communities, whereas antibiotic-treatment had little to no effect on the pulmonary microbiota but ameliorated the gut microbiota. Thus, while some neutrophilia lowering effects by hBD2 may be microbiota mediated, the highly similar effect sizes on lung neutrophilia produced by both hBD2 and antibiotics hint at a similar target beyond microbiota modulation.

Consistent with previous studies^6,34–38^, we show that CS has a profound impact on intestinal morphology and gut barrier dysfunction, impairing mucus production and key barrier markers. CS greatly impaired mucus production, a feature aggravated by antibiotic treatment. In contrast, hBD2 administration mitigated most CS-induced detrimental changes, even in antibiotic treated mice, indicating a mode of action dependent on direct hBD2–host interactions. As increased intestinal permeability could potentially impact the tissue distribution of orally delivered hBD2, and since it has previously been shown that direct intranasal delivery of hBD2 can limit pulmonary neutrophilia after CS exposure^21^, we measured hBD2 levels in colon tissue, serum, and lungs. Our LC-MS analyses did not yield any measurable traces of hBD2 outside the colon, in which we only detected low amounts aligning with our dosing regimen. As such we posit that the effects of oral hBD2 on the pulmonary environment must be due to local intestinal actions.

It is widely accepted that the mucus barrier is imperative to prevent gut bacteria and other luminal contents from encroaching the host epithelium and inducing pro-inflammatory responses^45,46^. Additionally, disruption of this intestinal firewall is associated with a wide range of both intestinal and extra-intestinal diseases such as inflammatory bowel diseases^47,48^ and metabolic diseases^49,50^. At the mucosal border, we show that CS triggers colonic bacterial encroachment and that antibiotics and hBD2 counteracted this, through distinct mechanisms. Antibiotic treatment ameliorated any gut bacterial signal, thus nullifying communication between host and microbe, despite greatly impairing mucus production and potentially decreasing distance from non-bacterial luminal content to the epithelium. In contrast, hBD2 preserved the gut microbiota and increased mucus abundance, greatly limiting bacterial encroachment. Surprisingly, few mice from the chronic RA-hBD2 group receiving antibiotics had, as the only antibiotics treated mice, higher bacterial abundance in the feces, supporting our observation of detectable bacteria in the PNA-FISH-stained tissue sections. Bacteria were here in direct contact with the intestinal epithelium. If dormant persister bacteria survived our antibiotic treatment, the dramatic encroachment might be explained by mucus defects induced by antibiotics, as also reported by a recent study elucidating how antibiotic treatment perturbs gut barrier function independent of gut microbiota eradication^39^. Antibiotics have been coupled to promotion of inflammation by facilitating bacterial translocation^46^ and repeated antibiotics usage disrupts the colonic mucus machinery considerably^40^, which is recapitulated in our study, despite the relative short-term (11 days) antibiotic treatment. Accumulating evidence suggests that this detrimental effect of antibiotics may be explained by direct, microbiota-independent effects on host colonocytes through ER stress, dysregulation of autophagy, and subsequent impairment of mucus production^39,51,52^.

Faulty mucus production may facilitate local gut inflammation, and thus signals relayed to the lungs – thereby aggravating irritant-induced pulmonary insults such as CS. To this end, the gut–lung axis has been increasingly appreciated over the last decade, with multiple studies highlighting the impact of the gut microbiota on pulmonary health. Despite this recent traction, findings across studies are not consistent. Seminal reports showed that germ-free mice presented increased pulmonary inflammation compared to conventional mice^4^, and that dietary fiber content and subsequent production of SCFAs dictated whether mice developed normal or aggravated inflammation during allergic airway challenge^5^, suggesting a protective role of the gut microbiota and its signaling molecules, at least in the context of allergic airway diseases. Interestingly, this seems to contrast the findings in COPD studies, where the gut microbiota appears to have a detrimental role, as studies, including ours, have found beneficial effects on pulmonary inflammation following antibiotic treatments^6,22^. One explanation for the discrepancy of the gut microbial role between studies of allergic airway disease and CS exposure may lie in the disease context. While allergic airway disease is confined to the lungs, CS has been shown to alter both the gut and lung microbiota after prolonged exposure^6,53–57^ in parallel to changes to the gut mucosa corroborated by this study. Additionally, CS has been shown to alter the host antimicrobial response^17^ with human COPD patients having lower sputum levels of HDPs, including hBD2^58^. The microbiota of COPD patients may therefore be more pro-inflammatory and have an altered metabolite pool, and host defenses may be compromised, allowing microbes to encroach the epithelium, as we demonstrate here, skewing host immunity toward a pro-inflammatory profile. Gut–lung communication may further be directed through metabolites or via migration of immune cells between these distant organs. For instance, in a model of Helminth infection, Huang et al.^13^ demonstrate that intestinal group 2 innate lymphoid cells (ILC2) are recruited to the lungs to mediate tissue repair after helminth larvae infiltrate lung tissues. Similarly, Pu and colleagues^14^ have also shown that migration of ILC2 occurs during inflammation, and that this process partly depends on the gut microbiota. ILC2 are known to accumulate in the lungs following induction of allergic airway disease independently of the method of disease induction^59^. Combined, this suggests that the gut microbiota, if tweaked correctly, may be beneficial in allergic airway disease while detrimental in CS-induced COPD.

Finally, it has previously been shown that intranasal installation of hBD2 following CS exposure impacts pulmonary neutrophil, macrophage, and dendritic cell populations^21^. As mucosal immune compartments are highly interconnected, it will be of immense importance for future studies to disentangle if the communications involved in the gut–lung axis in the context of CS exposure are due to direct host immune–microbe interactions and immune cell trafficking between gut and lungs, or through circulating metabolites.

In conclusion, we provide compelling evidence strengthening the importance of the gut–lung axis in COPD. CS greatly impairs colonic mucus, allowing gut microbes to encroach the epithelial layer, potentially increasing the pro-inflammatory interactions between the host and its microbes. Intriguingly, limiting host–microbe interactions at the gut mucosae in this context reduces the inflammatory milieu of the airways. Future studies should investigate if these effects are mediated through direct host immune-microbe interactions or changes to signaling molecules distributed between the gut and the lungs, during CS exposure, just as any potential sex-differences in either disease penetrance or treatment efficacy should be further elucidated. Given that chronic antibiotic administration to COPD patients would not be ethical due to the rising antimicrobial crisis and the negative impact of antibiotics on gut health, identifying innovative and safe supplement strategies strengthening the mucosal barrier and reversing the CS-induced mucus deterioration could potentially serve as a novel treatment strategy for patients with CS-induced lung inflammation and even COPD.

## Methods

### Laboratory animals, cigarette smoke exposure, and hBD2 administration

Animal protocols were approved by the Committee on the Ethics of Animal Experiments of Université Laval (no 2022–1126). Animal experiments were performed using 6- to 8-week-old female C57BL/6NCrl mice from Charles River Laboratories (Montréal, Canada). Mice were maintained according to guidelines from the Guide for the Care and Use of Laboratory Animals of the Canadian Council on Animal Care (CCAC) and housed five/cage in IVC cages at 12 h/12 h dark/light cycle with *ad libitum* access to standard rodent chow and water. Mice exposed to cigarette smoke (CS) using a whole-body exposure system (SIU24; Promech Lab AB, Vintrie, Sweden) using 24 cigarettes with filters removed (3R4F reference cigarettes; University of Kentucky, Lexington, KY, USA), or control room air (RA), with exposure duration depending on the protocol. In the acute protocol, mice were exposed to RA or CS for 2 hours every morning for four consecutive days, whereas in the chronic protocol, mice were exposed to RA or CS for 5 days/week for 7 weeks. Two hours after CS exposure vehicle control (sterile water) or hBD2 (Novozymes, Denmark) was delivered by oral gavage (2.4 mg/kg, in 200 µL of sterile water) daily in the acute protocol, or during the *last 4 days* of CS exposure in the long-term 7-week chronic exposure protocol. To assess the role of an intact microbiota, mice were randomly allocated to either a control drinking water or an antibiotics group, in which the latter were given a broad-spectrum antibiotics cocktail of 0.5 g/L Neomycin (Sigma #N1876-25 G) and 1 g/L Ampicillin (Sigma #A9518-25 G) in their drinking water for 11 days total – 7 days prior to administration and sustained during the 4 days of administration. Fecal samples were collected at baseline longitudinally until termination. Mice were anesthetized with isoflurane and euthanized by exsanguination of the descending aorta following retro-orbital blood collection.

### Bronchoalveolar lavage and differential cell count

Open-chested bronchoalveolar lavages were performed as follows: lungs were removed from the thoracic cavity, the trachea was cannulated, and the right lobes were tied off. The left lobe as lavaged once with 250 µl PBS and again with 200 µl. Non-lavaged lobes were collected, immediately frozen in liquid nitrogen, and kept at −80°C until RNA extraction or pulmonary microbiota analysis. Total cell counts were performed on the BAL using a hemocytometer. The BAL was then centrifuged (800 xg, 10 min, 4°C), and the BAL fluid was kept at −80°C. Cells were resuspended in PBS and cytospins were prepared and stained using the Hema 3 protocol (Fisher) for differential cell counts. A total of 300 cells were counted per cytospin per mouse using ImageJ software.

### Pulmonary macrophage size

Pulmonary macrophage size was quantified using the ImageJ Software as follows: using the ImageJ Grid Plugin, the surface area of 30 randomly selected macrophages per cytospin per mouse was measured and the mean size expressed as a total pixel covered area and/or percentage of the mean macrophage size of control room air-exposed mice.

### Blood sampling

Blood samples were taken from mice deeply sedated under isoflurane via retro-orbital bleeding. Immediately after blood collection, each sample was kept at room temperature for 1 hour before centrifugation (12000 g, 10 min, 4ºC). The serum phase was collected, and samples were stored at −80ºC.

### Sampling of intestinal tissue

Sections of duodenum, jejunum, ileum, and colon tissue were dissected from the intestine of each animal, the weight of the sections documented and then snap frozen immediately in liquid nitrogen and stored at −80°C. Duodenum was considered the first 5 cm of the small intestine. For the remaining small intestine tissue, the first 3 cm were discarded, and the remaining of the proximal 2/3 of the small intestine were categorized as jejunum. The first 3 cm of the distal 1/3 of the small intestine was discarded and the remaining tissue categorized as ileum. Prior to sectioning the colon, its length was measured. Cecum was weighed, and cecal content was snap frozen immediately in liquid nitrogen and stored at −80°C.

### Mucus preservation and bacterial encroachment

One cm pieces of distal ileum, proximal colon, and distal colon were dissected and put in Methacarn Fixative (60% v/v anhydrous methanol, 30% v/v chloroform, 10% v/v gl. acetic acid) and stored at room temperature for 24 h and subsequently stored at 4°C until processing. For five random mice per group per experimental protocol, a universal bacterial double-labeled TexasRed-conjugated peptide nucleic acid (PNA)-immunofluorescent *in situ* hybridization (FISH) probe^60,61^ was used to stain the bacteria at the surface of the intestinal mucosa. In short, Methacarn fixated tissues were washed in methanol for 2 × 30 min, ethanol for 2 × 15 min, ethanol/TissueClear (1:1) 1 × 15 min, and TissueClear for 2 × 15 min followed by paraffin embedding. The tissue sections were deparaffinized in 2 × 5 min xylene, followed by 2 × 3 min 99% ethanol, 2 × 3 min 96% ethanol, and 2 × 3 min sterile Milli-Q water. The PNA-FISH probe was applied on the sections, protected by cover slides, and incubated at 55℃ for 90 min. Excess staining was removed by incubation in wash buffer (AdvanDx, Woburn, MA, US) at 55℃ for 30 min, and the sections were counterstained with 4’,6-diamidino-2-phenylindole (DAPI) (Life Technologies, Oregon, USA) (3 µM) for 15 min, rinsed with Milli-Q water, and left to airdry. Once dry, mounting medium was applied (Prolong Gold, Life technologies, Oregon, USA), and the cover slides were sealed with clear nail polish. During the cutting and staining process, surfaces and tools were carefully cleaned to avoid contamination with bacterial DNA from the environment.

Image acquisition was done by confocal laser scanning microscopy (CLSM) with settings according to Ring et al. 2017 using a LSM980 (Zeiss, Jena, Germany) with an EC Plan-Neofluar 10x/0.3 (numerical aperture) air and a Plan-Apochromat 63 x/1.4 oil objective (Zeiss). For each mouse, three representative 1.8255·10^4^ µm^2^ areas from the proximal colon were captured and the distances between the microbiota and colonic epithelium were measured using the Image-Pro Premier 9.2 software (Media Cybernetics). Graphs of bacterial encroachment include individual data point representing each mouse. In short, the space between microbiota and epithelium was highlighted in the Image-Pro Premier 9.2 software, and the software performed between 100 and 200 distance measurements within the highlighted area. Thus, the data is reported as the average of 300–600 measurements per mouse. PNA-FISH images represent the median of each group and include the highlighted areas from which distances were calculated.

### Histological scoring of intestinal tissue

Intestinal morphology and mucus area were blindly assessed using PAS-stained slides. Crypt depth (CD) of proximal colon samples was assessed by measuring the depth of three crypts per subject, only when the entire crypt epithelium was visible from the *lamina muscularis mucosa* to the intestinal lumen. Mucus area was analyzed using the Image-Pro Premier 9.2 software by quantifying PAS^+^ color staining within three full-length adjacent crypts, in addition to the total crypt area for a mucus:crypt area ratio, repeated three separate places within each section; thus, each datapoint represents the average of PAS^+^ color staining of 9 crypts per mouse. CDs were analyzed using the Zeiss Zen Desk Software, from three separate areas within each section and reported as the average of measurements. Digital images were acquired using the Zeiss Axioscan Z.1 (Zeiss, Jena, Germany).

### Bacterial DNA extraction

Bacterial DNA was extracted using the MN Stool 96 (Machery Nagel) and MN Soil kit (Machery Nagel) with centrifuge processing. The purified DNA was quantified using a Nanodrop 2000 (Thermo Scientific). All samples were processed within 1 week and stored at −20°C and analyzed within 1 week.

### Quantification of fecal bacterial load

Quantification of the bacterial 16S rRNA gene was performed by qPCR using a Light Cycler 480 II (Roche). The 16S rRNA gene V4 region-specific Primers 505F (5’-GTGYCAGCMGCCGCGGTAA-3’) and 806 R (5’-GGACTACNVGGGTWTCTAAT-3’) were chosen for quantifying the relative gene abundance of all bacteria. A two-fold standard curve was produced by serially diluting the DNA pool (starting dilution: 1:20) in sterile MilliQ Water. DNA was diluted 20–150-fold depending on DNA concentration, to fall within the standard curve. The qPCR reaction was based on the SYBR™ Green Universal Master Mix from Applied Biosystems using 4 µL diluted DNA and a final concentration of 500 nM of each primer in a total volume of 10 µL. The thermal cycling conditions started with a DNA-denaturation step at 95°C for 5 min, followed by 50 cycles of (i) denaturation at 95°C for 10 s, (ii) annealing at 60°C for 20 s, and (iii) extension at 72°C for 20 s. All samples were run in triplicates. It was ensured that data from each triplicate fell within the standard curve and significant outliers (>2% CV) were excluded before calculating the average Ct of each sample. As a proxy for bacterial abundance, qPCR threshold cycle (Ct) values were converted to estimated bacterial genomes present in 1 mg of feces.

### RT-qPCR analysis of relative gene expressions)

#### cDNA synthesis

Reverse transcription was performed according to protocol for SuperScriptTM IV VILO Master Mix (Thermo Fischer). In brief, 2 µg of total RNA was mixed with 4 µL SuperScriptTM IV VILO Master Mix on ice and the total volume was adjusted to 20 µL by Nuclease-free water. The reverse transcription was performed in the PCR thermo-cycler using a predefined program: Primer annealing: 25°C for 10 min; Reverse transcription: 50°C for 10 min; Enzyme inactivation: 85°C for 5 min. All cDNA preparations were stored at −80°C. Working solutions were prepared by diluting the cDNA 30 times (20 µL cDNA +580 µL H_2_O).

#### Quantitative PCR (qPCR)

qPCR was performed in 384-well plates. For each reaction, cDNA was pre-diluted 1:1 in Nuclease free H_2_O and 3 µL cDNA was added to respective wells in 384-well plate. A polymerase enzyme Master mix was prepared in 200 µL tube. For one reaction ( = 7 µL/reaction) the following was mixed: TaqMan Fast Advanced Master Mix: 5 µL + TaqMan Assay (20x) (Primer) ( = 0.5 µL/reaction). The Master Mix was adjusted to a total volume of 7 µL by 1.5 µL Nuclease free H_2_O. The Master mix was scaled up according to the number of samples incl. controls included in the set-up. Each well in the 384-well plate was loaded with 7 µL Master Mix and 3 µL prediluted cDNA. The plate was sealed and spun to ensure sedimentation, mix of cDNA, and Master Mix.

The qPCR was performed in the QuantStudio 5.0 PCR apparatus using the “Comparative Ct Fast” set-up in a predefined program: Polymerase activation: 95°C for 20 min. (1 cycle) followed by 40 cycles of denaturation: 95°C for 1 min. and annealing/extension: 60°C for 20 sec.

All qPCR data were analyzed by delta delta Ct analysis (2−ΔΔCt) using the QuantStudio 5.0 software. The housekeeping gene *Eef2* was used for normalization and RA-Vehicle-H_2_O mice were used as reference point.

### 16S rRNA gene amplicon sequencing

DNA was extracted following the manufactures protocol for the NucleoSpin soil kit (Macherey-Nagel). The V3V4 region of the bacterial 16S rRNA gene was amplified by quantitative PCR (qPCR) using the 341.2FDI and 805.2RDI primers described in Larsen et al. inspired by Klindworth ^62^ with a 5’ 8nt barcodes arranged as 12 × 24 combinatorial barcoding for 384 well plate format inspired by^63^. The PCR mix contained 1 µl 5x Platinum II Taq Hot-Start buffer (Thermo Fisher, MA, USA), 0.05 µl 50 mm MgCl2, 0.07 µl 25 mm dNTP mix, 0.01 µl SYTO-16 (Thermo Fisher, MA, USA), 0.04 µl Platinum II Taq Hot-Start DNA Polymerase (Thermo Fisher, MA, USA), 0.83 µl PCR grade water, 1 µl 0.5 µM primer mix, and 2 µl DNA template. The PCR was performed on a QuantStudio 7 Flex (Thermo Fisher, MA, USA) with initial denaturation for 2 min at 95°C followed by 30 cycles of denaturation for 15 sec at 95°C, annealing for 30 sec at 55°C and extension for 1 min at 68°C. All PCR reactions from a given 384 well plate were pooled equi-volumetrically for a final volume of 1920 µl and added 300 µl EDTA (0.5 M). Three hundred microliters of this pool was purified with AMPure XP beads using a 1:1 ratio following manufactures’ protocol (Beckman Coulter, CA, USA). Illumina compatible adaptors were added to 417 ng of the purified amplicon following the TagSteady protocol^64^. The amplicon library was sequenced on an Illumina NextSeq 1000 (Illumina, CA, USA) in 300 bp paired end mode.

### 16S rRNA gene amplicon sequence analysis

The UPARSE pipeline was used to generate zero radius operational taxonomic units (OTUs) as described in^42^. The taxonomical annotation of unique filtered OTUs was conducted using the feature-classifier classify-sklearn in the Qiime2 v2024.5 framework, where a reference databased was trained on the Greengenes2 database v2022.10^65^.

### Tissue hBD2 distribution and quantification

Samples were homogenized using a bead beater (Bertin) and extracted in solvent (80% MeOH, 10% HCOOH, 500 mg/L benzalkonium chloride) under cold conditions. Then samples were centrifuged and transferred to plastic HPLC vials before analysis. The hBD2 concentrations were quantified against an in-house hBD2 standard using liquid chromatography-tandem mass spectrometry (LC-MS/MS) using a Xevo TQ-S micro (Waters) with Acquity UPLC.

#### LC-MS/MS settings

Mobile phase A: 0.15% formic acid in water and mobile phase B: 0.15% formic acid in acetonitrile. The flow rate was 0.25 mL/min and gradient elution was used: 0–1 min: 88% A, 1–4 min: gradient to 55% A, then to 5% in 0.5 min, followed by an isocratic wash with 5% A. The total method run time was set to 10 min including equilibration. The column temperature was 60°C and the sample temperature was 4–10°C. The injection volume was 5 µL for both samples and standards. A standard curve of HBD2 from 2.7 to 333 µg/L was generated in sample preparation solvent. The Xevo TQ-S micro was operated in ESI+ mode with the following settings: Voltage: 3 kV, Cone: 16 V, Desolvation temp: 350°C, Desolvation gas flow: 500 L/hr, cone gas flow: 20 L/min, source temp: 150°C, MRM setting: 866.65 >> 143.11 Da using collision energy at 52 V.

### Serum and lung lipocalin-2 quantification by ELISA

Levels of lipocalin-2 (LCN2) were quantified using the LCN2/NGAL specific MSD U-PLEX kit from Meso Scale Diagnostics following the manufacturer’s instructions.

### Statistical analyses

Statistical analyses were performed using GraphPad Prism (version 10). When comparing multiple groups, and data were normally distributed, each plot shows the p-value and asterisk from Two-Way ANOVA followed by Tukey’s multiple comparisons post-hoc test. An overview of all statistical analyses can be found in Supplemental_Material_1.

#### Beta diversity

Beta diversity was assessed using Weighted UniFrac distance matrices, which account for both the phylogenetic relationships between taxa and their relative abundances. To evaluate the effects of experimental factors on beta diversity, we performed a permutational multivariate analysis of variance (PERMANOVA) using the adonis2 function from the vegan R package. While PERMANOVA tests the differences in group centroids, it is sensitive to differences in dispersion. To test if statistical differences were due to centroid differences or group dispersion, we used the betadisper function from the vegan package. The resulting p-values were adjusted for multiple comparisons where necessary, and an alpha level of 0.05 was used as the threshold for significance.

#### Differentially abundant taxa

Non-rarefied feature tables were processed using the DESeq2 framework with default settings to identify differentially abundant taxa. The DESeq2 workflow involved the following steps: (1) estimation of size factors, which normalize library sizes in a model-based fashion to account for differences in sequencing depth, (2) estimation of dispersions for each taxon, and (3) fitting of each taxon based on the specified experimental groupings, with hypothesis testing performed using the default Wald test. Differential abundance results were obtained using the results function, which provided Benjamini–Hochberg false discovery rate (FDR)-adjusted p-values and log2 fold-change effect sizes. Taxa were considered significantly differentially abundant if they met the significance threshold of FDR-adjusted p-value <0.01 and had an absolute log2 fold change ≥2. These data were visualized using volcano plots, with the log2 fold change on the x-axis and the -log10-transformed adjusted p-value on the y-axis.

## Supplementary Material

Supplemental_Material_1.xlsx

## Data Availability

All data are available at Mendeley data (https://data.mendeley.com/datasets/ph3dkb947t/1). OTU tables are deposited with informative sample ID, and raw data from each figure panel is compiled in an Excel metafile with clearly labeled sheets. Each sheet contains information about project ID, cage ID, mouse ID, treatment, and experimental day.
